# Evolution ventilator associated pneumonia (VAP) in our intensive care unit, after pneumonia zero (PZ) program implementation

**DOI:** 10.1186/2197-425X-3-S1-A709

**Published:** 2015-10-01

**Authors:** R Fernández Fernández, ME Yuste Ossorio, MR Ramírez Puerta, O Moreno Romero, M Muñoz Garach

**Affiliations:** Hospital Universitario San Cecilio, Unidad Cuidados Intensivos, Granada, Spain

## Introduction

The National Survey of Nosocomial Infection Surveillance (ENVIN) is a computerized record of the incidence of nosocomial infection in ICU in Spain. Most international scientific societies have developed their own recommendations to avoid VAP. In our hospital, we have drafted rules for the maintenance of the airway in mechanically ventilated patients following general recommendations (PZ).

## Objectives

We want to describe our program and the evolution of our rates after its implementation and compare it with Spanish data.

## Methods

• Data from the ENVIN registration: 01.04.11 / 01.04.15. 682 hospital beds, 18 ICU beds.

• Conduct training and educational campaign (presencial and by means of internet) to professionals of ICU also including anesthesia-resuscitation unit (total of 112 people) with online assessment.

• Pharmacy Service prepared chlorhexidine (2%) oral solution for oral cleaning, we asked for orotracheal tubes and tracheostomy cannulas subglottic suctioning, tubing and active humidification steam and created a hand hygiene group ...Were instituted mandatory measures for the prevention of VAP:proper training in airway management, strict hand hygiene, control of pneumopressure ball(> 20 cmH_2_O), oral hygiene every 6-8 hours, avoid, whenever possible, supine position at 0 °, reduce intubation and / or its duration, prevent the programated change of tubing, humidifiers and tracheal tubes

- Statistical Support ENVIN: Rate/100 patients admitted, 100 patients mechanical ventilation (pMV), incidence density (ID)/1000 days of stay (ds), 1000 MV days (dMV). N = 4198 pattients admitted, 805 pMV. 17575 ds, 6087 dMV

## Results

**01.04.11-01.04.12**: 15 VAP. Rates: 1.46/admitted, 7.5/pMV. ID: 3.1/ ds,8.73/dMV Germ: A. baumanii 44.44%. Severe sepsis 33.33%. Sepsis 33.33%.

**01.04.12-01.04.13**: 6 VAP. Rates: 0.55/admitted, 3.03/pMV. ID: 1.22/ds, 3.26/dMV

Germ: S. aureus 22.22%, S. maltophilia 22.22%. Sepsis 50%

**01.04.13-01.04.14**: 10 VAP. Rates: 0.89/admitted, 4.65/pMV. ID: 2.04/ds, 6.11/dMV

Germ: S. aureus 25%, E. coli 16.67%. Sepsis 50%

**01.04.14-01.04.15**: 4 VAP. Rates: 0.42/admitted, 2.07/pMV. ID: 0.95/ds, 2.71/dMV

Germ: H.influenzae 25%, K.oxytoca 25%, P.aeuroginosa 25%, S. liquefaciens 25%.

Severe sepsis 50%. Sepsis 50%.

**Spain** last year: Rates: 2.48/admitted, 5.92/pMV. ID: 3.28/ds, 6.87/dMV. Germ: P.aeuroginosa 21.1%. Sepsis 56.4%

## Conclusions

Our PZ program, has very good results, we are below Spanish data and ENVIN target: “ rate < 9/1000 dMV”[1]. In 2013, we presented an increase in the rate of NAV in connection with lower adherence to recommendations. In the last year we have managed to reduce to less than half the number of NAV. This is due to increased monitoring of adherence to these recommendations. Sepsis is the most frecuent response. Germs vary each year.
